# Evaluation of an attenuated chicken-origin *Histomonas meleagridis* vaccine for the prevention of histomonosis in chickens

**DOI:** 10.3389/fvets.2024.1491148

**Published:** 2024-11-25

**Authors:** Qiao-Guang Chen, Ling-Ming Kong, Jie Rong, Chen Chen, Shuang Wang, Zhao-Feng Hou, Dan-Dan Liu, Jian-Ping Tao, Jin-Jun Xu

**Affiliations:** ^1^College of Veterinary Medicine, Yangzhou University, Yangzhou, China; ^2^Jiangsu Co-innovation Center for Prevention and Control of Important Animal Infectious Diseases and Zoonosis, Yangzhou, China

**Keywords:** *Histomonas meleagridis*, Histomonosis, attenuation, chicken, vaccination

## Abstract

**Introduction:**

Histomonosis, a protozoan disease caused by *Histomonas meleagridis*, poses a significant economic burden on domestic poultry in China. To reduce the losses caused by this disease in chickens, an attenuated vaccine was developed by exploiting the diminished virulence of *H. meleagridis* through successive *in vitro* passages.

**Methods:**

Four experiments were conducted to evaluate the viability of attenuated *H. meleagridis* as a potential vaccine candidate. Experiment 1 evaluated the route of infection (oral vs. intracloacal) and dose (5 × 10^4^, 1 × 10^5^, and 2 × 10^5^*H. meleagridis*/chicken) using the virulent strain *H. meleagridis* JSYZ-D10. Experiment 2 evaluated the attenuated effect of the *H. meleagridis* JSYZ-D168 strain (infection dose: 2 × 10^5^*H. meleagridis*/chicken). Experiment 3 evaluated the immunoprotective effect of different immunization doses (5 × 10^4^, 1 × 10^5^, and 2 × 10^5^*H. meleagridis*/chicken). Experiment 4 evaluated the immunoprotective effect of different immunization schedules (immunization at 3 days of age; immunization at 14 days of age; two immunizations, one at 3 days of age and one at 14 days of age; immunization and infection dose: 2 × 10^5^*H. meleagridis*/chicken).

**Results:**

The results showed that the intracloacal route of infection was more effective and stable compared to the oral route. The pathogenicity of the JSYZ-D168 *H. meleagridis* strain was significantly reduced compared to the original virulent strain. Chickens vaccinated by intracloacal immunization at a dose of 2 × 10^5^*H. meleagridis*/chicken on day 14 provided effective protection against a virulent strain challenge, significantly resulting in increased body weight and reduced lesions in the cecum and liver within 28 days post-immunization (*p* < 0.05). Poor immunoprotection was obtained either when the immunization dose was 1 × 10^5^*H. meleagridis*/chicken or when the immunization program was a single immunization at 3 days of age only.

**Discussion:**

In conclusion, the administration of a vaccine provides a measurable degree of protection against the detrimental effects induced by *H. meleagridis*, thus warranting its endorsement in clinical settings.

## Introduction

Histomonosis is a protozoan disease of poultry caused by *Histomonas meleagridis* (1). The typical lesions are characterized by thickening of the cecal wall, cecitis, and circular crateriform necrosis on the liver (2). Since 2015, the morbidity and mortality of turkeys have been as high as 80 to 100 per cent due to the prohibition of nitarsone and lack of new preventive drugs approved for the treatment (3, 4). No candidate compounds able to replace the once used nitroimidazole and arsenic compounds have yet emerged (5, 6). The absence of effective pharmaceuticals has resulted in economic losses for producers, including increased mortality rates among turkeys and diminished production performance in both broilers and laying hens (7, 8). There exists a pressing imperative to identify alternative methods, beyond pharmaceutical interventions, for the management of histomonosis.

Attempts at active immunization through intramuscular injection of inactivated *H. meleagridis* or passive immunization via antisera injection into native poultry have failed to confer protection against virulent challenges (9, 10). Although IgG levels increased in birds following infection with virulent *H. meleagridis*, they do not appear to play a substantial role in development of protective immunity (11, 12). Consequently, the exploration of live vaccines capable of inducing cellular immunity has become a major focus in the field of immunoprophylaxis.

The earliest development of live attenuated vaccines can be traced back to Tyzzer in experiments of inconsistent reductions in virulence of *H. meleagridis* when propagated continuously *in vitro* over extended periods, leading to a loss of pathogenicity to chickens. However, it was noted that inducing protection against virulent strains was only achievable through propagation in the chicken cecum (13). Subsequent findings revealed that *H. meleagridis* lost both its pathogenic capacity and immune protective efficacy when the number of passages was too high (14). Prolonged *in vitro* passages decrease vaccine efficacy, but serial passage in poultry restores attenuated *H. meleagridis* to its original virulence (15). In experimental settings, oral or intracloacal inoculation of attenuated *H. meleagridis* provided some protection against virulent challenge and reduced liver and cecal lesions in chickens and turkeys (9, 16). Following prolonged *in vitro* passage, *H. meleagridis* may lose its ability to invade other host tissues and can be observed only in cecal tissue (17). Eighteen-week-old pullets vaccinated with the attenuated strain did not experience a severe drop in egg production When later infected with the virulent strain (16). Additionally, studies have shown that the attenuated strain provides a degree of cross-protective against heterologous virulent strains (18, 19). Although the recovery of virulence following consecutive passages in poultry has been recognized, some studies have demonstrated a lack of virulence recovery after 295 consecutive passages *in vitro*, followed by 5 subsequent *in vivo* reverse passages (9, 20).

In China, while the number of turkeys is relatively small, various breeds of chickens are raised in large quantities (21). DNA cloning and sequencing of the diseased livers from affected chickens revealed that the 18S rRNA gene of *H. meleagridis* in Jiangsu, China, shared 98.8–99.8% homologous with the French strain and 99.1–99.8% homologous with the Austrian strain, while the *β*-tubulin gene showed 94.9–97.1% homologous with the German strain and 91.6–93.7% homologous with the U.S. strain (22, 23). A recent epidemiological survey indicated that Chinese local laying hens and Chinese San Huang broilers were the most susceptible to histomonosis, with an average morbidity of 27.4%, of which 86.9% were in chickens less than 3 months old (8). As a result, our laboratory conducted the isolation, culture, and passaging of chicken-origin *H. meleagridis* for attenuation. Building on this, the present study aimed to investigate the protective effects of *in vitro* passaged chicken-origin *H. meleagridis* on chickens under experimental conditions and to further enrich the relevant data.

## Materials and methods

### Source of experimental animals

One-day-old Jinghai yellow chickens (JH chickens) were obtained from a local commercial hatchery (Haimen Street, Haimen District, Nantong, Jiangsu, China) and housed in a strictly sanitized animal facility of Yangzhou University. Adequate feed and water were provided (no medication was added to the feed and water). All animal handling procedures were complied with the regulations of the animal ethics committee of Yangzhou University (ethical review no. 202103210).

### *Histomonas meleagridis* isolate and culture

*H. meleagridis*, named JSYZ-D, was isolated from the liver of infected chickens in Yangzhou and preserved in parasite Laboratory of the school of veterinary medicine, Yangzhou University (24). The original strain was selected as the vaccine strain after 168 serial passages *in vitro*. Low passage (<10 serial passages) original strain (JSYZ-D 10) were used for virulent strain in all experiments. Parasites were passaged every 3 days *in vitro*. Anaerobic incubation at 40°C using standard culture flasks (T25, 25cm^2^ flask). The standard medium is composed of Medium 199 (Gibco, California, USA) and 10% inactivated horse serum (Gibco), 11 milligrams of sterilized rice starch (Sigma-Aldrich, ShangHai, China), with a total volume of 10 mL (25). At the time of the first isolation and culture of *H. meleagridis*, cecal bacteria (Cecal bacterial species were identified as *Escherichia coli* and *Klebsiella pneumoniae* by microbial mass spectrometry) were isolated from the cecal contents of healthy chicken using fresh blood agar medium and added to the co-culture, and not thereafter (19, 26). 10% dimethyl sulfoxide (sigma Aldrich, Shanghai, China) was used as cryoprotectant for long-term preservation. The number of *H. meleagridis* cells/ml was calculated by hemocytometer and trypan blue (Sigma-Aldrich, ShangHai, China) staining.

### Comparison of optimal infection dose and route (Experiment 1)

This trial aimed to evaluate the optimal route and dose of artificial infection.

Seventy chickens, reared in steel cages with wire flooring were weighed and randomly divided into seven groups at 14 days of age (adjustments were made to ensure that the average weight of each group was similar): Oral 50 k, Oral 100 k, Oral 200 k, Intracloacal 50 k, Intracloacal 100 k, Intracloacal 200 k, Non-challenge (NC). Each group was housed in a separate cage. On the day of grouping, the chickens in groups Oral 50 k, Oral 100 k, and Oral 200 k were orally inoculated with JSYZ-D10 at doses of 5 × 10^4^, 1 × 10^5^, and 2 × 10^5^ *H. meleagridis*/chicken, respectively (Fasting for 6 h before infection or 5 h post infection); the chickens in groups Intracloacal 50 k, Intracloacal 100 k, and Intracloacal 200 k were intracloacally inoculated with the JSYZ-D10 a at doses of 5 × 10^4^, 1 × 10^5^, and 2 × 10^5^ *H. meleagridis*/chicken, respectively; the chickens in group NC served as negative control (unchallenged with virulent strain). On day 28 (14 days post-challenge), individual body weights were recorded. All remaining chickens were euthanized, after which cecal and liver were assessed for lesion scoring (Table 1). Chickens that died during the experiment were immediately dissected and scored for lesions, and DNA from the liver and cecum were extracted for PCR detection (27).

**Table 1:** Experimental design.

Group	Number of chicken	Challenge strain	Challenge route	Challenge age (d)	Dose/chicken	End age (d)
Experiment 1						
Oral 50 k	10	D10	Oral	14	5.0 × 10^4^	28
Oral 100 k	10	D10	Oral	14	1.0 × 10^5^	28
Oral 200 k	10	D10	Oral	14	2.0 × 10^5^	28
Intracloacal 50 k	10	D10	Intracloacal	14	5.0 × 10^4^	28
Intracloacal 100 k	10	D10	Intracloacal	14	1.0 × 10^5^	28
Intracloacal 200 k	10	D10	Intracloacal	14	2.0 × 10^5^	28
NC	10	–	–	–	–	28
Experiment 2						
D10	10	D10	Intracloacal	14	2.0 × 10^5^	28
D168	10	D168	Intracloacal	14	2.0 × 10^5^	28
NC	10	–	–	–	–	28

Group	Number of chicken	Immunization age (d)	Immunization strain	Immunization does/chicken	Immunization route	Challenge age (d)	Challenge strain	Challenge dose/chicken	Challenge route	End age (d)
Experiment 3										
Vacc Intracloacal 50 k	10	14	D168	5.0 × 10^4^	Intracloacal	28	D10	2.0 × 10^5^	Intracloacal	42
Vacc Intracloacal 100 k	10	14	D168	1.0 × 10^5^	Intracloacal	28	D10	2.0 × 10^5^	Intracloacal	42
Vacc Intracloacal 200 k	10	14	D168	2.0 × 10^5^	Intracloacal	28	D10	2.0 × 10^5^	Intracloacal	42
PC	10	–	–		Intracloacal	28	D10	2.0 × 10^5^	Intracloacal	42
NC	10	–	–			–		–	–	42
Experiment 4										
d3 Vacc	10	3	D168	2.0 × 10^5^	Intracloacal	28	D10	2.0 × 10^5^	Intracloacal	42
d14 Vacc	10	14	D168	2.0 × 10^5^	Intracloacal	28	D10	2.0 × 10^5^	Intracloacal	42
d3/14 Vacc	10	3,14	D168	2.0 × 10^5^	Intracloacal	28	D10	2.0 × 10^5^	Intracloacal	42
PC	10	–	–		Intracloacal	28	D10	2.0 × 10^5^	Intracloacal	42
NC	10	–	–			–		–	–	42

Oral 50 k, oral infection at a dose of 5 × 10^4^ JSYZ-D10 *H. meleagridis*/chicken; Oral 100 k, oral infection at a dose of 1 × 10^5^ JSYZ-D10 *H. meleagridis*/chicken; Oral 200 k, oral infection at a dose of 2 × 10^5^ JSYZ-D10 *H. meleagridis* /chicken; Intracloacal 50 k, intracloacal infection at a dose of 5 × 10^4^ JSYZ-D10 *H. meleagridis* /chicken; Intracloacal 100 k, intracloacal infection at a dose of 1 × 10^5^ JSYZ-D10 *H. meleagridis* /chicken; Intracloacal 200 k, intracloacal infection at a dose of 2 × 10^5^ JSYZ-D10 *H. meleagridis* /chicken; NC, non-challenged control. Experiment 2 group: D10, intracloacal infection at a dose of 2 × 10^5^ JSYZ-D10 *H. meleagridis*/chicken; D168, intracloacal infection at a dose of 2 × 10^5^ JSYZ-D168 *H. meleagridis*/chicken; NC, non-challenged control. Experiment 3 group: Vacc Intracloacal 50 k, intracloacal immunization at a dose of 5 × 10^4^ JSYZ-D168 *H. meleagridis*/chicken; Vacc Intracloacal 100 k, intracloacal immunization at a dose of 1 × 10^5^ JSYZ-D168 *H. meleagridis*/chicken; Vacc Intracloacal 200 k, intracloacal immunization at a dose of 2 × 10^5^ JSYZ-D168 *H. meleagridis*/chicken; PC, positive-challenged control; NC, non-challenged control. Experiment 4 group: d3 Vacc, intracloacal immunization at a dose of 2 × 10^5^ JSYZ-D168 *H. meleagridis*/chicken at 3 days of age; d14 Vacc, intracloacal immunization at a dose of 2 × 10^5^ JSYZ-D168 *H. meleagridis*/chicken at 14 days of age; d3/14 Vacc, intracloacal immunization at a dose of 2 × 10^5^ JSYZ-D168 *H. meleagridis*/chicken at 3 and 14 days of age, respectively; PC, positive-challenged control; NC, non-challenged control.

### Evaluation of attenuation effect of passaged strain (Experiment 2)

The purpose of this test was to assess whether the *H. meleagridis* JSYZ-D168 strain has been attenuated.

Thirty chickens reared in steel cages with wire flooring were weighed and randomly divided into three groups at 14 days of age: D168, D10, NC. Each group was housed in a separate cage. On the day of grouping, the chickens in group D168 were intracloacally inoculated with JSYZ-D168 at doses of 2 × 10^5^ *H. meleagridis*/chicken; the chickens in group D10 were intracloacally inoculated with JSYZ-D10 at doses of 2 × 10^5^ *H. meleagridis*/chicken; the chickens in group NC served as negative control (unchallenged with any strain). On day 28 (14 days post-challenge), individual body weights were recorded. All remaining chickens were euthanized, after which cecal and liver were assessed for lesion scoring (Table 1).

### Determination of optimal immune dose (Experiment 3)

The purpose of this test is to assess the optimal immunizing dose.

Fifty chickens reared in steel cages with wire flooring were weighed and randomly divided into five groups at 14 days of age: Positive challenge (PC), NC, Vacc Intracloacal 50 k, Vacc Intracloacal 100 k, Vacc Intracloacal 200 k. Each group was housed in a separate cage. On the day of grouping, the chickens in group Vacc Intracloacal 50 k were intracloacally inoculated with JSYZ-D168 at doses of 5 × 10^4^ *H. meleagridis*/chicken; the chickens in group Vacc Intracloacal 100 k were intracloacally inoculated with JSYZ-D168 at doses of 1 × 10^5^ *H. meleagridis*/chicken; the chickens in group Vacc Intracloacal 200 k were intracloacally inoculated with JSYZ-D168 at doses of 2 × 10^5^ *H. meleagridis*/chicken. Virulent infection was performed on day 28 (2 weeks after immunization), the chickens in groups Vacc Intracloacal 50 k, Vacc Intracloacal 100 k, Vacc Intracloacal 200 k and PC were inoculated intracloacally at doses of 2 × 10^5^ JSYZ-D10 *H. meleagridis*/chicken. On day 42 (14 days post-challenge), individual body weights were recorded. All remaining chicks were euthanized, after which cecal and liver were assessed for lesion scoring (Table 1).

### Screening for optimal immunization procedures (Experiment 4)

The purpose of this test is to compare the effects of different immunization programs.

Fifty chickens reared in steel cages with wire flooring were weighed and randomly divided into five groups at 3 days of age: PC, NC, d3 Vacc, d14 Vacc, d3/14 Vacc. Each group was housed in a separate cage. On the day of grouping, the chickens in group d3 Vacc and d3/14 Vacc were intracloacally inoculated with JSYZ-D168 at doses of 2 × 10^5^ cells/chicken. On day 14, chickens in group d14 Vacc and d3/14 Vacc were intracloacally inoculated with JSYZ-D168 at doses of 2 × 10^5^ *H. meleagridis*/chicken. Virulent infection was performed on day 28, the chickens in groups d3 Vacc, d14 Vacc, d3/14 Vacc and PC were intracloacally inoculated with JSYZ-D10 at doses of 2 × 10^5^ *H. meleagridis*/chicken. On day 42 (14 days post-challenge), individual body weights were recorded. All remaining chicks were euthanized, after which cecal and liver were assessed for lesion scoring (Table 1).

### Lesion scoring rules

Cecal scoring criteria (28, 29) were as follows: the longitudinal fold of the cecal wall was well characterized and lacked macroscopical lesions, and the cecal contents were thick with dark feces and no caseous exudate, score 0; cecal wall thickening or presence of scattered petechiae, or both, score1; moderate thickening of the cecal wall with caseous exudate or contents forming a caseous core, color change of cecal contents or absence of contents and bleeding spots in the cecum, score 2; the cecal wall was thickened, with a prominent caseous core of cecal contents, or the cecum had no contents or the cecal wall appeared petechiae, score 3; the wall of the cecum is significantly thickened, and the cecal mucosal layer appears fibrotic necrotic and ulcerated, with a caseous core or no contents in the cecum, the presence of a hemorrhagic blind end, or cecal rupture leading to peritonitis, score 4. Liver scoring criteria (28, 29) were as follows: no macroscopic round necrotic lesion, score 0; presence of 1–5 small round necrotic foci (< 5 mm in diameter), score 1; many small round necrotic foci (≥ 5), or large necrotic foci (≥ 5 mm in diameter), score 2; many macroscopic small and large necrotic foci, score 3; presented with complex lesions and numerous mixed lesions, score 4. All lesions were scored without knowing the grouping.

### Data processing and analysis

The experimental results were evaluated by morbidity (Check the chickens daily for any clinical signs suggestive of histomonosis such as loss of appetite, listlessness and feather disturbance), survival rate, body weight gain (BWG), relative weight gain rate (BWG of experimental group or PC group/BWG of NC group × 100%) and lesion scores of the liver and cecum. Differences in morbidities and each subgrouping of positive liver or positive cecal LS were compared to the NC group using the chi-square test. The data were statistically analyzed using one-way ANOVA by SPSS 22.0 (IBM SPSS, Inc., Chicago, IL, USA) with Duncan’s multiple range test. Data were expressed as mean ± S.D. value. Differences were considered significant at *p* < 0.05.

## Results

### Experiment 1

**Figure 1:**
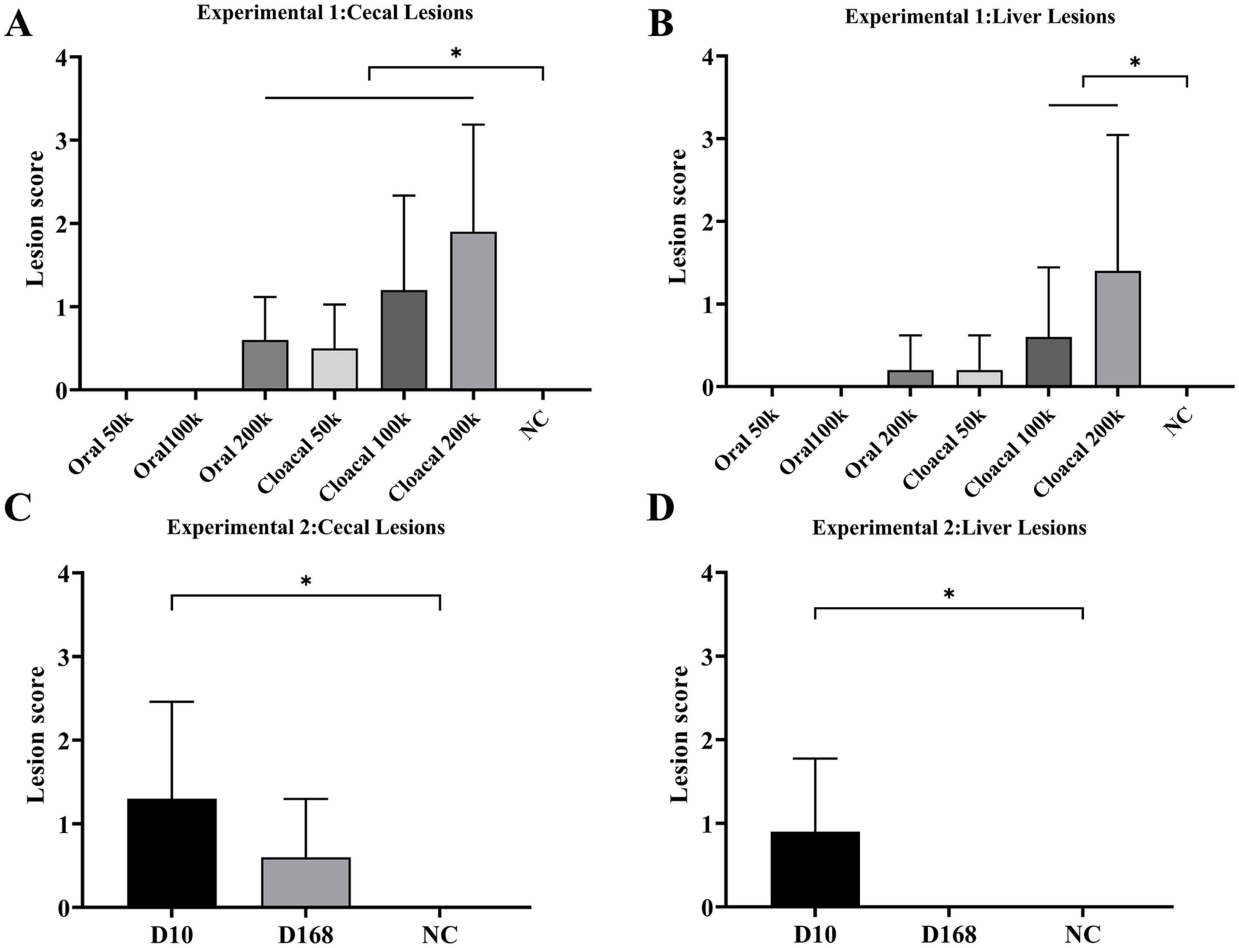
Experiment 1 Mean lesion scores for **(A)** cecae and **(B)** liver. “*” indicates significant difference between groups (*p* < 0.05). Abbreviations: Oral 50 k, oral infection at a dose of 5 × 10^4^ JSYZ-D10 *H. meleagridis*/chicken; Oral 100 k, oral infection at a dose of 1 × 10^5^ JSYZ-D10 *H. meleagridis*/chicken; Oral 200 k, oral infection at a dose of 2 × 10^5^ JSYZ-D10 *H. meleagridis* /chicken; Intracloacal 50 k, intracloacal infection at a dose of 5 × 10^4^ JSYZ-D10 *H. meleagridis* /chicken; Intracloacal 100 k, intracloacal infection at a dose of 1 × 10^5^ JSYZ-D10 *H. meleagridis* /chicken; Intracloacal 200 k, intracloacal infection at a dose of 2 × 10^5^ JSYZ-D10 *H. meleagridis* /chicken; NC, non-challenged control. Experiment 2 Mean lesion scores for **(C)** cecae and **(D)** liver. “*” indicates significant difference between groups (p < 0.05). Abbreviations: D10, intracloacal infection at a dose of 2 × 105 JSYZ-D10 *H. meleagridis*/chicken; D168, intracloacal infection at a dose of 2 × 105 JSYZ-D168 *H. meleagridis*/chicken; NC, non-challenged control.

3/10, 5/10 and 6/10 chickens in the oral 200 k, intracloacal 100 k and intracloacal 200 k groups, respectively, showed symptoms of listlessness, feather disturbance and excretion of sulfur like feces post infection. The morbidity of oral 50 k, oral 100 k, oral 200 k, intracloacal 50 k, intracloacal 100 k and intracloacal 200 k groups were 0, 0, 30, 0, 50 and 60%, respectively. On the 13th day post infection, one chicken died in the intracloacal 200 k group. The survival rate of intracloacal 200 k group was 90%, and that of the other groups was 100%. The body weight and body weight gain of the Oral 200 k, intracloacal 100 k, and intracloacal 200 k groups exhibited a significant decrease compared to the NC group (*P* < 0.05). Conversely, the body weight and body weight gain of the remaining groups did not display a significant difference when compared to the NC group (*P* > 0.05) (Table 2). In all groupings, those with a positive rate of liver lesions for the Oral 50 k, Oral 100 k and Oral 200 k groups were 0, 0, and 20% while those with a positive rate of cecal lesions were 0, 0, and 60%, respectively. Those with a positive rate of liver lesions for the Intracloacal 50 k, Intracloacal 100 k and Intracloacal 200 k groups were 20, 40, and 50% while those with a positive rate of cecal lesions were 40, 70, and 90%, respectively. The positive rate of both liver and cecum in the NC group was 0%. The mean lesion score of liver and cecum in the Intracloacal 200 k group was significantly higher than those in the NC group (*P* < 0.05). There was no difference between the other groups and the NC group (*P* > 0.05) (Table 3, Figures 1A,B, Supplementary Figure S1).

**Table 2:** Weight change in each group.

Group	Morbidity (%)	Survival rate (%)	Weight before infection (g)	Weight post infection (g)	Body weight gain (g)	Relative weight gain (%)
Experimental 1						
Oral 50 k	0	100	183.3 ± 6.1^a^	473.3 ± 12.3^a^	290.0 ± 14.1^a^	96.7
Oral 100 k	0	100	183.3 ± 6.9^a^	471.2 ± 13.0^a^	287.9 ± 14.0^a^	96
Oral 200 k	30	100	183.3 ± 5.9^a^	452.7 ± 17.6^b^	269.4 ± 14.2^b^	89.9
Intracloacal 50 k	0	100	183.2 ± 7.0^a^	471.6 ± 14.6^a^	288.4 ± 13.3^a^	96.2
Intracloacal 100 k	50*	100	183.2 ± 5.9^a^	451.4 ± 23.0^b^	268.2 ± 21.2^b^	89.5
Intracloacal 200 k	60*	90	183.2 ± 6.4^a^	415.8 ± 18.6^c^	232.6 ± 20.6^c^	77.6
NC	0	100	183.4 ± 5.7^a^	483.1 ± 15.8^a^	299.6 ± 13.1^a^	100
Experimental 2						
D10	80*	100	183.1 ± 6.1^a^	421.1 ± 26.7^b^	237.8 ± 25.7^b^	74.9
D168	20	100	183.1 ± 5.5^a^	482.1 ± 21.2^a^	298.9 ± 20.1^a^	94.2
NC	0	100	183.2 ± 4.8^a^	500.3 ± 18.4^a^	317.1 ± 19.3^a^	100
Experimental 3						
Vacc Intracloacal 50 k	70*	100	181.4 ± 5.8^a^	764.4 ± 19.5^b^	582.8 ± 18.7^b^	92.7
Vacc Intracloacal 100 k	20	100	181.5 ± 4.7^a^	769.1 ± 16.2^b^	587.4 ± 16.5^b^	93.4
Vacc Intracloacal 200 k	20	100	181.7 ± 5.8^a^	785.3 ± 47.6^ab^	603.6 ± 44.3^ab^	96.0
PC	80*	100	181.6 ± 4.9^a^	686.2 ± 36.3^c^	504.7 ± 34.4^c^	80.3
NC	0	100	181.8 ± 4.0^a^	810.1 ± 30.4^a^	628.3 ± 28.5^a^	100
Experimental 4						
d3 Vacc	40	100	40.4 ± 0.8^a^	744.9 ± 28.6^b^	704.5 ± 28.4^b^	94.8
d14 Vacc	30	100	40.4 ± 1.0^a^	755.0 ± 25.1^ab^	714.6 ± 24.6^ab^	96.2
d3/14 Vacc	40	100	40.0 ± 0.8^a^	755.9 ± 24.8^ab^	715.9 ± 24.5^ab^	96.3
PC	70*	100	40.4 ± 0.9^a^	637.1 ± 45.8^c^	596.6 ± 45.7^c^	80.3
NC	0	100	40.6 ± 1.0^a^	783.5 ± 27.3^a^	742.8 ± 28.0^a^	100

*Indicates significant difference in morbidities (*P* < 0.05) as compared to the NC group with chi-square test. Values of body weight data within a column with no common superscript differ significantly (*p* < 0.05). Experiment 1 group: Oral 50 k, oral infection at a dose of 5 × 10^4^ JSYZ-D10 *H. meleagridis*/chicken; Oral 100 k, oral infection at a dose of 1 × 10^5^ JSYZ-D10 *H. meleagridis*/chicken; Oral 200 k, oral infection at a dose of 2 × 10^5^ JSYZ-D10 *H. meleagridis* /chicken; Intracloacal 50 k, intracloacal infection at a dose of 5 × 10^4^ JSYZ-D10 *H. meleagridis* /chicken; Intracloacal 100 k, intracloacal infection at a dose of 1 × 10^5^ JSYZ-D10 *H. meleagridis* /chicken; Intracloacal 200 k, intracloacal infection at a dose of 2 × 10^5^ JSYZ-D10 *H. meleagridis* /chicken; NC, non-challenged control. Experiment 2 group: D10, intracloacal infection at a dose of 2 × 10^5^ JSYZ-D10 *H. meleagridis*/chicken; D168, intracloacal infection at a dose of 2 × 10^5^ JSYZ-D168 *H. meleagridis*/chicken; NC, non-challenged control. Experiment 3 group: Vacc Intracloacal 50 k, intracloacal immunization at a dose of 5 × 10^4^ JSYZ-D168 *H. meleagridis*/chicken; Vacc Intracloacal 100 k, intracloacal immunization at a dose of 1 × 10^5^ JSYZ-D168 *H. meleagridis*/chicken; Vacc Intracloacal 200 k, intracloacal immunization at a dose of 2 × 10^5^ JSYZ-D168 *H. meleagridis*/chicken; PC, positive-challenged control; NC, non-challenged control. Experiment 4 group: d3 Vacc, intracloacal immunization at a dose of 2 × 10^5^ JSYZ-D168 *H. meleagridis*/chicken at 3 days of age; d14 Vacc, intracloacal immunization at a dose of 2 × 10^5^ JSYZ-D168 *H. meleagridis*/chicken at 14 days of age; d3/14 Vacc, intracloacal immunization at a dose of 2 × 10^5^ JSYZ-D168 *H. meleagridis*/chicken at 3 and 14 days of age, respectively; PC, positive-challenged control; NC, non-challenged control.

**Table 3:** Cecal and liver lesion rate and lesion scores.

Group	+Liver LS	+Cecal LS	Liver LS					Cecal LS				
			0	1	2	3	4	0	1	2	3	4
Experiment 1												
Oral 50 k	0/10 (0%)	0/10 (0%)	10	0	0	0	0	10	0	0	0	0
Oral 100 k	0/10 (0%)	0/10 (0%)	10	0	0	0	0	10	0	0	0	0
Oral 200 k	2/10 (20%)	6/10 (60%)*	8	2	0	0	0	4	6	0	0	0
Cloacal 50 k	2/10 (20%)	4/10 (40%)	8	2	0	0	0	5	5	0	0	0
Cloacal 100 k	4/10 (40%)	7/10 (70%)*	6	2	2	0	0	3	4	1	2	0
Cloacal 200 k	5/10 (50%)*	9/10 (90%)*	5	1	3	0	1	1	4	1	3	1
NC	0/10 (0%)	0/10 (0%)	10	0	0	0	0	10	0	0	0	0
Experiment 2												
D10	6/10 (60%)*	7/10 (70%)*	4	3	3	0	0	3	3	2	2	0
D168	0/10 (0%)	5/10 (50%)*	10	0	0	0	0	5	4	1	0	0
NC	0/10 (0%)	0/10 (0%)	10	0	0	0	0	10	0	0	0	0
Experiment 3												
Vacc Cloacal 50 k	2/10 (20%)	6/10 (60%)*	8	2	0	0	0	4	4	2	0	0
Vacc Cloacal 100 k	1/10 (10%)	4/10 (40%)	9	1	0	0	0	6	3	1	0	0
Vacc Cloacal 200 k	0/10 (0%)	4/10 (40%)	10	0	0	0	0	6	4	0	0	0
PC	8/10 (80%)*	9/10 (90%)*	2	7	1	0	0	1	6	2	1	0
NC	0/10 (0%)	0/10 (0%)	10	0	0	0	0	10	0	0	0	0
Experiment 4												
d3 Vacc	2/10 (20%)	5/10 (50%)*	8	2	0	0	0	5	3	2	0	0
d14 Vacc	1/10 (10%)	4/10 (40%)	9	1	0	0	0	6	3	1	0	0
d3/14 Vacc	0/10 (0%)	4/10 (40%)	10	0	0	0	0	6	4	0	0	0
PC	4/10 (40%)	8/10 (80%)*	6	1	3	0	0	2	3	3	2	0
NC	0/10 (0%)	0/10 (0%)	10	0	0	0	0	10	0	0	0	0

^*^Indicates significant difference in morbidities (*P* < 0.05) as compared to the NC group with chi-square test. Experiment 1 group: Oral 50 k, oral infection at a dose of 5 × 10^4^ JSYZ-D10 *H. meleagridis*/chicken; Oral 100 k, oral infection at a dose of 1 × 10^5^ JSYZ-D10 *H. meleagridis*/chicken; Oral 200 k, oral infection at a dose of 2 × 10^5^ JSYZ-D10 *H. meleagridis* /chicken; Intracloacal 50 k, intracloacal infection at a dose of 5 × 10^4^ JSYZ-D10 *H. meleagridis* /chicken; Intracloacal 100 k, intracloacal infection at a dose of 1 × 10^5^ JSYZ-D10 *H. meleagridis* /chicken; Intracloacal 200 k, intracloacal infection at a dose of 2 × 10^5^ JSYZ-D10 *H. meleagridis* /chicken; NC, non-challenged control. Experiment 2 group: D10, intracloacal infection at a dose of 2 × 10^5^ JSYZ-D10 *H. meleagridis*/chicken; D168, intracloacal infection at a dose of 2 × 10^5^ JSYZ-D168 *H. meleagridis*/chicken; NC, non-challenged control. Experiment 3 group: Vacc Intracloacal 50 k, intracloacal immunization at a dose of 5 × 10^4^ JSYZ-D168 *H. meleagridis*/chicken; Vacc Intracloacal 100 k, intracloacal immunization at a dose of 1 × 10^5^ JSYZ-D168 *H. meleagridis*/chicken; Vacc Intracloacal 200 k, intracloacal immunization at a dose of 2 × 10^5^ JSYZ-D168 *H. meleagridis*/chicken; PC, positive-challenged control; NC, non-challenged control. Experiment 4 group: d3 Vacc, intracloacal immunization at a dose of 2 × 10^5^ JSYZ-D168 *H. meleagridis*/chicken at 3 days of age; d14 Vacc, intracloacal immunization at a dose of 2 × 10^5^ JSYZ-D168 *H. meleagridis*/chicken at 14 days of age; d3/14 Vacc, intracloacal immunization at a dose of 2 × 10^5^ JSYZ-D168 *H. meleagridis*/chicken at 3 and 14 days of age, respectively; PC, positive-challenged control; NC, non-challenged control.

### Experiment 2

The morbidity of D168 and D10 groups were 20 and 80%, respectively. The survival rate of every group was 100%. The body weight and body weight gain of D10 group were significantly lower than those of NC group (*P* < 0.05). The body weight and body weight gain of the D168 group were not significantly different from those of the NC group (*P* > 0.05) (Table 2). The positive rate of liver lesions for the D10 and D168 groups were 60 and 0% while those with a positive rate of cecal lesions were 70 and 50%, respectively. Compared with the NC group, the liver lesion score and the cecal lesion score in the D10 group were significantly increased (*P* < 0.05), while there was no significant difference in the liver lesion score and the cecal lesion score in the D168 group (*P* > 0.05). The liver lesion score and cecal lesion score were significantly lower in the D168 group than in the D10 group (*P* < 0.05) (Table 3, Figures 1C,D, Supplementary Figure S2).

### Experiment 3

**Figure 2:**
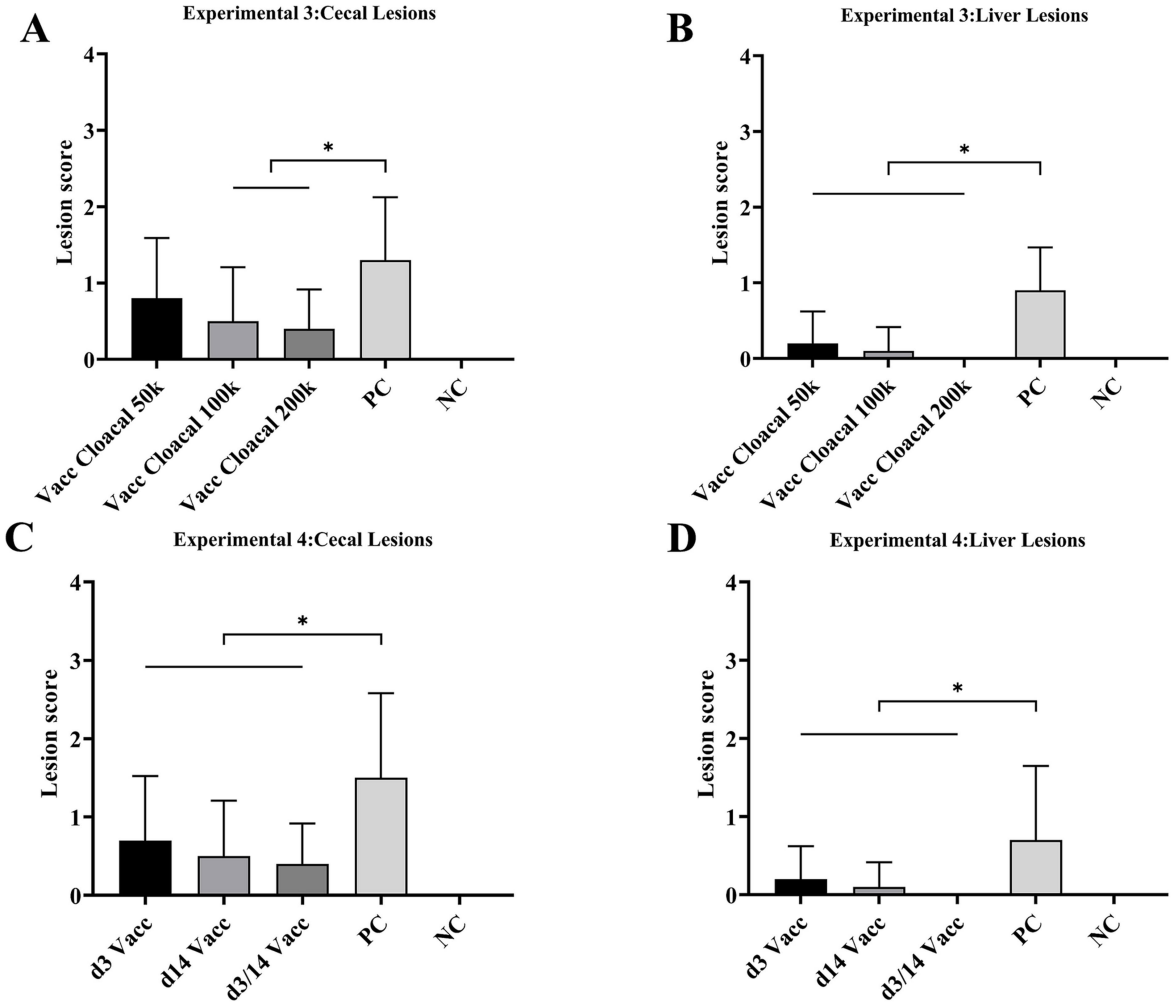
Experiment 3 Mean lesion scores for **(A)** cecae and **(B)** liver. “*” indicates significant difference between groups (*p* < 0.05). Abbreviations: Vacc Intracloacal 50 k, intracloacal immunization at a dose of 5 × 10^4^ JSYZ-D168 *H. meleagridis*/chicken; Vacc Intracloacal 100 k, intracloacal immunization at a dose of 1 × 10^5^ JSYZ-D168 *H. meleagridis*/chicken; Vacc Intracloacal 200 k, intracloacal immunization at a dose of 2 × 10^5^ JSYZ-D168 *H. meleagridis*/chicken; PC, positive-challenged control; NC, non-challenged control. Experiment 4 Mean lesion scores for **(C)** cecae and **(D)** liver. “*” indicates significant difference between groups (*p* < 0.05). Abbreviations: d3 Vacc, intracloacal immunization at a dose of 2 × 10^5^ JSYZ-D168 *H. meleagridis*/chicken at 3 days of age; d14 Vacc, intracloacal immunization at a dose of 2 × 10^5^ JSYZ-D168 *H. meleagridis*/chicken at 14 days of age; d3/14 Vacc, intracloacal immunization at a dose of 2 × 10^5^ JSYZ-D168 *H. meleagridis*/chicken at 3 and 14 days of age, respectively; PC, positive-challenged control; NC, non-challenged control.

The morbidity of vacc intracloacal 50 k, vacc intracloacal 100 k, vacc intracloacal 200 k and PC groups were 70, 20, 20 and 80%, respectively. The survival rate of every group was 100%. The d42 body weight and mean weight gain of the Vacc Intracloacal 50 k, Vacc Intracloacal 100 k and Vacc Intracloacal 200 k group were significantly higher than those of the PC group (*P* < 0.05). However, there were still significant differences between groups Vacc Intracloacal 50 k and Vacc Intracloacal 100 k on d42 weight and mean weight gain compared with the NC group (*P* < 0.05), and no significant differences between Vacc Intracloacal 200 k and the NC group (*P* > 0.05) (Table 2). In all groups, the positive rate of liver lesions for the vacc intracloacal 50 k, vacc intracloacal 100 k and vacc intracloacal 200 k groups were 20, 10, and 0% while those with a positive rate of cecal lesions were 60, 40, and 40%, respectively. The mean liver lesion score showed that there was no significant difference between the Vacc Intracloacal 50 k, Vacc Intracloacal 100 k, Vacc Intracloacal 200 k and the NC group (*P* > 0.05), while these four groups differed significantly compared to the PC group (*P* < 0.05). The mean cecal lesion score showed that the NC group were significantly lower than the Vacc Intracloacal 50 k group (*P* < 0.05), while the PC group was significantly higher than the Vacc Intracloacal 100 k, Vacc Intracloacal 200 k and the NC groups (*P* < 0.05). There was no significant difference in liver lesion score or cecal lesion score among different immune groups (*P* > 0.05), but numerically, the Vacc Intracloacal 200 k group was the most effective (Table 3, Figures 2A,B, Supplementary Figure S3).

### Experiment 4

The morbidity of d3 Vacc, d14 Vacc, d3/14 Vacc and PC groups were 40, 30, 40 and 70%, respectively. No chickens died, and the survival rate of every group was 100%. The body weight and body weight gain were significantly higher in the d3 Vacc, d14 Vacc and d3/14 Vacc groups than in the PC group (*p* < 0.05). There was no significant difference in body weight and body weight gain in the d14 Vacc and d3/14 Vacc groups compared to the NC group (*p* > 0.05), while the difference was significant in the d3 Vacc group (*p* < 0.05). There was no significant difference in body weight and body weight gain among the d3 Vacc, d14 Vacc and d3/14 Vacc group (*p* > 0.05), but from the data of body weight gain, d14 Vacc group and d3/14 Vacc group were better than d3 Vacc group (Table 2). The positive rate of liver lesions for the d3 Vacc, d14 Vacc and d3/14 Vacc groups were 20, 10, and 0% while those with a positive rate of cecal lesions were 50, 40, and 40%, respectively. The mean liver lesion score showed that the d3 Vacc, d14 Vacc and d3/14 Vacc groups were significantly lower than the PC group (*p* < 0.05), and had no significant difference from the NC group (*p* > 0.05). The mean cecal lesion score showed that the d3 Vacc, d14 Vacc and d3/14 Vacc groups were significantly lower than the PC group (*p* < 0.05), and also had no significant difference from the NC group (*p* > 0.05). There were no significant differences between the d3 Vacc, d14 Vacc and d3/14 Vacc groups in either the mean liver lesion score or the mean cecal lesion score (*p* > 0.05), with the d3/14 Vacc group having the lowest value in terms of lesion score (Table 3, Figures 2C,D, Supplementary Figure S4).

## Discussion

Histomonosis is an economically significant poultry disease, primarily affecting turkeys, and is found across all continents (30). With no alternative drugs available for treatment and prevention, vaccination provides a safe and residue-free strategy to protecting poultry (3, 5). China hosts a significant diversity of avian species and holds the largest population and production scale of yellow broiler worldwide (8). The prevalence of histomonosis, a serious epidemic, requires urgent attention due to the significant threat it poses (8, 21). In this study, *H. meleagridis* isolated from chickens was used, and local poultry breeds were selected for testing to obtain results more reflective of actual production conditions.

In existing reports, the primary mode of experimental infection with *H. meleagridis* mainly contains two types, oral infection and intracloacal infection (9, 31). Based on the findings of experiment 1 of this study, while oral infection is more convenient than intracloacal infection, its efficiency is relatively low. Significantly more chickens in the intracloacally infected group exhibited symptoms of histomonosis compared to the orally infected group, with the only death occurring in the intracloacal 200 k group. The Oral 50 k and Oral 100 k groups were not affected by the infection, as indicated by body weight gain and lesion scoring results. Liver and cecum lesions, as well as weight loss, were observed only in the oral 200 k group. However, in the intracloacal 50 k, intracloacal 100 k and intracloacal 200 k groups, varying degrees of liver and cecum lesions and weight loss were observed. The efficiency of oral infection is largely influenced by the acidic environment of the avian stomach (29, 32). Studies have shown that fasting prior to oral infection or feeding turkeys an alkaline mixture can increase the severity of infection lesions (29). In this study, fasting prior to oral infection was also employed; however, the findings indicated significantly lower efficacy compared to turkeys. Specifically, while a dose of 1 × 10^4^ *H. meleagridis*/turkey was sufficient for successful infection in turkeys, the present study necessitated a higher infection dose of 2 × 10^5^ *H. meleagridis*/chicken to ensure oral infection (29, 31, 33). Successful infection of chickens with a dose of 1 × 10^4^ *H. meleagridis* would require both oral and intracloacal routes (34). This study established the optimal infection route for the artificial infection model and identified the most suitable route for live vaccine immunization of JH chickens. Given that these differences may be due to variations among avian species, subsequent trials all used the intracloacal inoculation method to achieve more consistent infection results (35–37).

In Experiment 2, the *H. meleagridis* JSYZ-D strain, after 168 times *in vitro* passages, caused only mild cecal pathological damage post infection and had a minimal impact on body weight. This aligns with previous studies in which the pathogenicity of *H. meleagridis* was attenuated by repeated passages *in vitro* (9, 17, 38). As the number of passages increases, the pathogenicity of *H. meleagridis* decreases, accompanied by a corresponding decline in immunogenicity (14). Previous research has demonstrated that the 95, 215, and 295 generations effectively protected turkeys from mortality following virulent *H. meleagridis* challenges post-immunization (9). It is crucial to note that fewer passages are associated with a greater impact of attenuated parasites on avian subjects (39), and typically, strains with more than 200 passages are selected for attenuation (9, 20, 40). However, due to the differing susceptibilities of chickens and turkeys to *H. meleagridis* (2, 37, 41), the D168 generation strain, which had been attenuated while remaining infectious, was chosen as a candidate strain for investigation in this study.

Oral vaccination (10^4^ *H. meleagridis*/turkey) of 1-day-old turkeys with *in vitro* attenuated *H. meleagridis* successfully prevented histomonosis (33). Similarly, intracloacal vaccination (10^4^ *H. meleagridis*/turkey) of 14-day-old turkey can also help resist *H. meleagridis* infection (9). However, based on the results of Experiment 1, the effect of low-dose (5 × 10^4^ *H. meleagridis*/chicken) infection in chickens was minimal for both oral and intracloacal routes of infection. Additionally, data from Experiment 3, the effect of low-dose immunization (5 × 10^4^ *H. meleagridis*/chicken) had no significant effect in preventing infection. Lesion scoring results from Experiment 3 indicated that an immunization dose of at least 10^5^ *H. meleagridis*/chicken is required for effective resistance to the virulent *H. meleagridis* infection. Furthermore, based on body weight variation, an immunization dose of 2 × 10^5^ *H. meleagridis*/chicken is required to achieve no significant difference from the negative control group (*p* > 0.05). The minimum immunization dose required for turkeys to achieve immunological protection is documented as 10^3^ cell per turkey (42). These variations in immunization dosage may be attributed to different methods of parasite attenuation, as well as the contrasting susceptibility of JH chickens and turkeys to *H. meleagridis* (36, 41, 42). Overall, a dose of at least 10^5^ *H. meleagridis*/chicken is required to elicit an effect, either by infection or immunization. Given the practical value of JH chickens, minimizing the impact on body weight is preferable, provided there is no mortality following infection.

In Experiment 4, chickens immunized at 3 days of age exhibited the lowest level of immune protection after being challenged with virulent *H. meleagridis*. However, the immune protection in chickens immunized at 14 day of age and in the 3/14-day secondary immunization group was nearly identical and their body weights did not differ significantly from the negative control group (*p* > 0.05). Notably, even in the 3-day immunized group, which had the lowest level of immunoprotection, the lesion scores of the cecum and liver were significantly lower than those of the positive control group (*P* < 0.05). In turkeys, immunization at 1 day of age is sufficient to help the host resist the invasion of the virulent strain, while immunization at 14 days of age or secondary immunization at 1/14 days of age more effectively mitigates the damage caused by virulent strain infections (19, 33, 42). Lesion scores visually indicate that organ damage in immunized chickens is primarily limited to the cecum, with few lesions observed in the liver. This phenomenon is observed in both chickens and turkeys (16). In terms of immunoprotection, both a single immunization at 14 days of age and two immunizations at 3/14 days of age demonstrate greater efficacy compared to a single immunization at 3 days of age, consistent with previous research findings (19). However, considering practical operational factors, prioritize a single immunization at 14 days of age is recommended.

Over the past two decades, approximately 15 strains have been used in studies on the immunoprophylaxis of *H. meleagridis*. The majority of these strains were derived from diseased turkeys, with only four originating from diseased chickens (18, 19, 43, 44). Geographically, four strains were found in the United States (10, 19, 45), one in the United Kingdom (44), two in France (18, 42), two in Germany (18, 41), one in Belgium (42), and five in Austria (12, 16, 18, 46, 47), with no reports from Asia. In terms of pathogenicity, there appears to be minimal variation in the pathological damage induced by the strains documented thus far, regardless of whether the strains originate from chickens or turkeys (18, 41). This observation is further supported by the findings of the present study. However, given the global prevalence of histomonosis, reports from any geographical region are significant for understanding and managing the disease (35, 48). This study represents the first vaccine evaluation trial conducted in Asia. Although a cross-immunoprotection trial was not feasible due to the unavailability of turkey-derived strains in the region, numerous studies have provided evidence supporting the reliability of cross-immunoprotection against *H. meleagridis* (18, 19, 47). In conclusion, this study tested the route, dose and procedure of immunization, addressing the data gap on vaccination against *H. meleagridis* in Asia and China. Additionally, it indirectly demonstrated the viability of vaccination as a key strategy for global *H. meleagridis* control. However, large-scale animal experiments were not conducted in this study, and further testing is still required.

## Data Availability

The original contributions presented in the study are included in the article/Supplementary material, further inquiries can be directed to the corresponding author.
