# Supplementary material for: Evaluation of an attenuated chicken-origin Histomonas meleagridis vaccine for the prevention of histomonosis in chickens
Source: Front Vet Sci. 2024 Nov 25;11:1491148. doi: 10.3389/fvets.2024.1491148 (PMC11625761; doi:10.3389/fvets.2024.1491148)
Supplement: Supplementary file 1 [file Supplementary_file_1.docx]

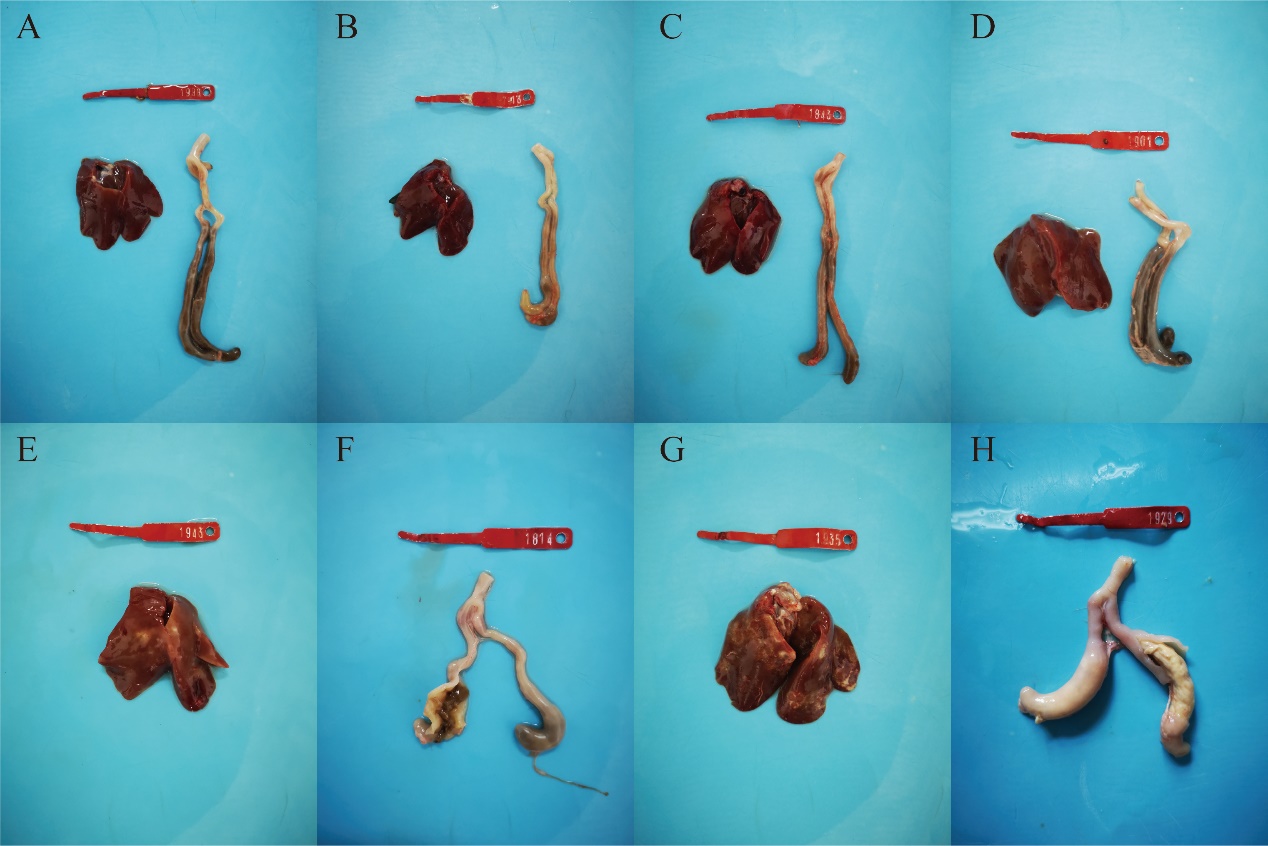


**Supplementary Figure 1.** Experiment 1 cecal and liver lesions in each group. A, liver and cecum in group Oral 50k; B, liver and cecum in group Oral 100k; C, liver and cecum in group Oral 200k; D, liver and cecum in group Cloacal 50k; E, Liver lesions in group Cloacal 100k; F, cecal lesions in group Cloacal 100k; G, Liver lesions in group Cloacal 200k; H, cecal lesions in group Cloacal 100k.
