# Supplementary material for: Evaluation of an attenuated chicken-origin Histomonas meleagridis vaccine for the prevention of histomonosis in chickens
Source: Front Vet Sci. 2024 Nov 25;11:1491148. doi: 10.3389/fvets.2024.1491148 (PMC11625761; doi:10.3389/fvets.2024.1491148)
Supplement: Supplementary file 2 [file Supplementary_file_2.docx]

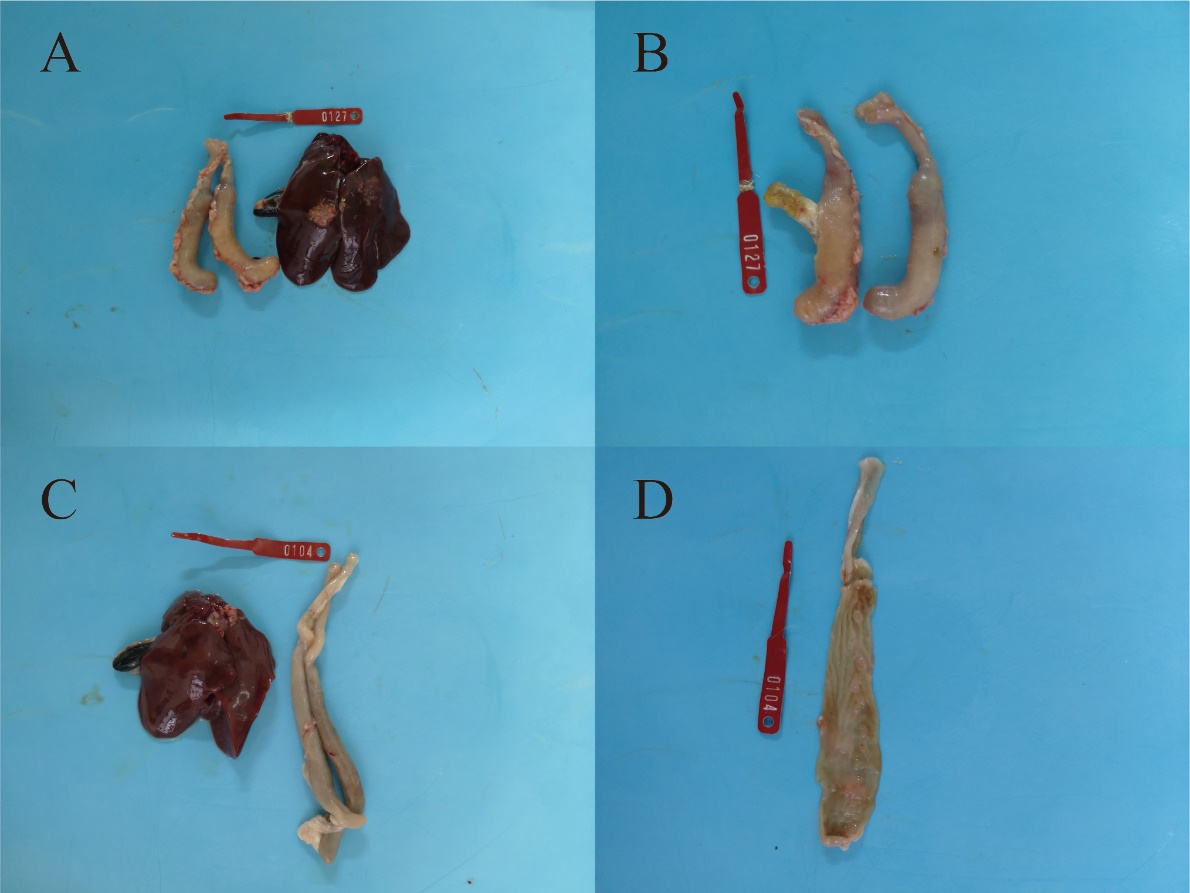


**Supplementary Figure 2.** Experiment 2 cecal and liver lesions in each group. A, liver and cecum in group D10; B, cecal lesions in group D10; C, liver and cecum in group D168; D, cecal lesions in group D168.
